# Scale-appropriate mechanization impacts on productivity among smallholders: Evidence from rice systems in the mid-hills of Nepal

**DOI:** 10.1016/j.landusepol.2019.03.030

**Published:** 2019-06

**Authors:** Gokul P. Paudel, Dilli Bahadur KC, Dil Bahadur Rahut, Scott E. Justice, Andrew J. McDonald

**Affiliations:** a International Maize and Wheat Improvement Center, Kathmandu, Nepal; b International Maize and Wheat Improvement Center, El Batan, Mexico

**Keywords:** Farm mechanization, Technology adoption, Impact assessment, Mini-tillers, Smallholder rice farmers, Endogenous switching regression, Agricultural productivity, South Asian hills

## Abstract

Smallholder farmers in the mid-hills of Nepal are facing an acute labor shortage due to out-migration which, in general, has affected the capacity to achieve timely crop establishment, harvest, and inter-cultural operations. These effects are more visible in the case of labor-intensive crops such as rice and promoting higher levels of rural mechanization has emerged as the primary policy response option. Nevertheless, quantitative evidence for the ability of mechanization to offset the adverse effects of shortages increasing labor prices in these systems is largely absent. This study investigates the impacts associated with adoption of mini-tillers (5 to 9 horsepower) for land preparation on smallholder rice productivity in the mid-hills of Nepal. We use an endogenous switching regression that accounts for both observed and unobserved sources of heterogeneity between mini-tiller adopters and non-adopters. Findings demonstrate that rising on-farm rural wage rates and an emerging decline in draft animal availability are driving adoption of the mini-tiller. Among users, the mini-tiller increased rice productivity by 1,110 kg/ha (27%). Further, regression results suggest that mini-tiller non-adopters would be able to increase their rice productivity by 1,250 kg/ha (26%) if they adopt. Moreover, our analysis revealed that very small farms (≤0.25 ha) that adopt mini-tillers are benefited the most in terms of gains in rice productivity. These findings support policies that favor the expansion of small-scale mechanization in the hill production ecologies of South Asia and highlight the need to foster the emergence of an associated service economy that will permit smallholders access to capital-intensive machinery such as the mini-tiller.

## Highlights

• Labor shortage due to out-migration adversely affected smallholder farmers in Nepal.
• Adoption of scale-appropriate mechanization increased rice productivity by 1110 kg/ha.
• Adoption of mechanization by non-adopters would increase rice productivity by 1250 kg/ha.
• Very small rice cultivating farms (≤0.25 ha) benefited most from adoption of mechanization.
• Rising on-farm rural wage due to labor shortage was the primary driver of mini-tiller adoption.

## 1 Introduction

The benefits of farm mechanization in the agriculture sector are well known (Kienzle et al., 2013; Pingali, 2007). In developing countries with small farms, low productivity, and widespread poverty, mechanization is particularly important to decrease the cost of production, improve farm efficiency, reduce drudgery, and improve crop productivity (Benin, 2015; Kienzle et al., 2013; Pingali, 2007; Sims and Kienzle, 2006). However, there exists an almost universal positive relationship between farm size and level of mechanization (Ghosh, 2010; Jha et al., 2000; Jha and Rhodes, 1999; Van Den Berg et al., 2007). Other factors that favor mechanization include land consolidation and market integration (Fan and Chan-Kang, 2005; Hazell, 2005; Otsuka et al., 2016; Rigg et al., 2016; Xiaobing et al., 2016; Yang et al., 2013). Nevertheless, mechanization levels are growing among smallholders in developing countries, indicating increased incentives and capacity to adopt scale-appropriate mechanization, particularly through service provision (Diao et al., 2014; Keil et al., 2016; Mottaleb et al., 2017, 2016; Yamauchi, 2016; Zhang et al., 2014). Policy makers have taken note and have identified mechanization as a core dimension required to support sector growth (e.g., Nepal’s Agriculture Development Strategy), but there is a dearth of studies that provide insights into the farm-level importance of mechanization in neglected production ecologies in areas such as the mid-hills of Nepal, hence policymakers are often making decision without an accurate understanding of the potential returns on public investment.

Nepal is an agricultural country where almost two-thirds of its population is engaged in agriculture, and agriculture contributes almost one-third of the national gross domestic product (MoAD, 2016). However, agriculture in Nepal is basically of a subsistence type, and the productivity of cereal crops is the lowest among the South Asian countries (FAO, 2019). Food insecurity is a major problem with more than two-thirds of the districts facing food shortages every year (Joshi et al., 2012). In recent years, Nepalese agriculture has experienced an accelerating trend of labor out-migration, particularly to middle-east countries in search of better job opportunities (Maharjan et al., 2013a). This has created acute labor shortages in the agriculture sector that have affected timely crop establishment and other crop cultivation practices (ILO, 2017; Maharjan et al., 2013b, 2013a).

This situation has contributed to rising rural wage rates (Wiggins and Keats, 2014; Wang et al., 2016). While the rising rural wage rates are desirable for agricultural workers, significantly negative impacts on farm enterprise profitability and productivity are common. Furthermore, despite persistent low-crop productivity and high food insecurity, the agricultural area remaining fallow due to rising labor prices in Nepal is increasing (Khanal, 2018; Khanal et al., 2015; Maharjan et al., 2013b; Prabakar et al., 2011). In this context, policy makers in Nepal have identified scale-appropriate mechanization as a vital component of agricultural sector growth as well as jobs creation.

In the mid-hills of Nepal, the rice productivity is lower due to delayed seedling transplantation resulting from extended time for land preparation due to labor shortage. However, adoption of scale-appropriate farm mechanization can help farmers prepare their field and transplant rice seedlings on time. Previous studies have also shown that rising labor scarcity and/or increase in labor wages as the major driver for adopting farm mechanization (Reddy et al., 2014; Wang et al., 2016; Win and Thinzar, 2016; Yang et al., 2013; Zhang et al., 2014). Furthermore, the labor scarcity during land preparation prolongs the age of rice seedlings and delayed transplanting of older seedlings can reduce effective panicles, tillers, and rice-grain yield significantly (Liu et al., 2017). Liu et al. (2015) reported that transplanting older seedlings can reduce rice productivity by 31%.

Over 90% of the rice in Nepal is transplanted manually using human labor, and animal traction (Upadhyaya, 1996) and in most of the villages, farmers start the land preparation at the same time (June-July), farmers cannot find adequate laborers and bullocks for the land preparation. Rice-land preparation using traditional bullocks and laborers takes 64 h per hectare, while the scale-appropriate farm mechanization can prepare the same land in approximately 20 h per hectare. Farmers who are unable to prepare land on time are compelled to transplant older seedlings which reduces rice productivity and thereby increases food insecurity (Kushwaha, 2018; Lampayan et al., 2015; Pasuquin et al., 2008). Hence, the adoption of scale-appropriate farm mechanization can support farmers to prepare their fields on time and increase rice productivity and food security.

## 2 Farm mechanization in Nepal

The history of farm mechanization in Nepalese agriculture dates back to the early 1970s with the introduction of four-wheeled tractors and later Japanese, Korean, and Chinese two-wheeled tractors in the 1980s (Biggs and Justice, 2015). The spread of these two-wheeled tractors (also known as power-tillers) continued until 2004, and the introduction of small-scale mechanization started in the country (Biggs et al., 2011; Biggs and Justice, 2015). Mechanization early on was usually only used for tractor tillage until the advent of threshers during the 1990s and combine-harvesters in the 2000s in Nepal Terai (Biggs et al., 2011).

**Fig. 1:**
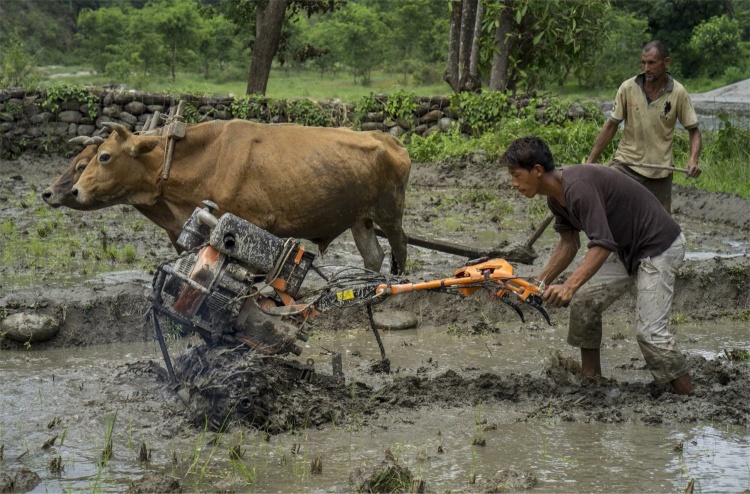
Farmers in the mid-hills of Nepal using mini-tiller and bullocks for rice field preparation in two different plots.

The four- and two-wheeled tractors were heavily concentrated in the large valleys and plains in the Terai region of Nepal (Gauchan and Shrestha, 2017). However, mechanization in the hills remained challenging due to difficult topography, fragmented land, and small and terraced plots. Takeshima (2017) reported that less than 8% of the farms used mechanized tillage in the hills and mountains, while 46% of the farms used mechanized tillage in the Terai, indicating the wide spatial heterogeneity in terms of farm mechanization in Nepal. In the Terai, mechanization is more pervasive due to the flatter topography and easy access to inputs, road networks, and the markets (Takeshima, 2017a, 2017b, Takeshima et al., 2017, 2015). In the hills mechanization started only after 2010, when Chinese small horsepower tractors (also known as mini-tillers: Fig. 1) came into the Nepalese market, although small-scale threshers and pump-sets were prevalent in selected hilly areas (Justice and Biggs, 2013).^1^

Although the government and some project-based demonstrations contributed, the private sector played a major role in bringing mini-tillers to Nepal primarily from manufacturers in China (Gauchan and Shrestha, 2017). In 2014, the Government of Nepal promulgated a farm mechanization policy which articulated several farm mechanization promotion policies, including the provision of subsidies for the mini-tillers (Gauchan and Shrestha, 2017; Takeshima, 2017a). Furthermore, in April 2015, a 7.8 magnitude earthquake struck the mid-hills of Nepal (Mitchell et al., 2017b), and almost 9000 people died, along with over 17,000 livestock - primarily the draft animals (GoN, 2015). In the aftermath of the earthquake, many development organizations distributed mini-tillers to farmers to offset the acute scarcity of labor and draft animals. These programs further accelerated the adoption of mini-tillers in the mid-hills of Nepal. Currently, over 10,000 mini-tillers are being used in the mid-hills of Nepal (CSISA, 2018). While the spread of mini-tillers in the mid-hills of Nepal continues, questions have emerged at the policy level about their contribution to food security and livelihoods. We use the case of rice in order to investigate the performance of mini-tiller adoption on rice productivity. Rice, as a primary staple crop of Nepal, is grown in almost 1.5 million hectares and the national average productivity is 3.4 tons per hectare (MoAD, 2016). Traditionally rice is transplanted in puddled fields, and such areas occupy over 90% of the rice area in the mid-hills of Nepal (Upadhyaya, 1996). Rice is considered a labor-intensive crop since it requires a large number of laborers for nursery establishment, seedling uprooting, tillage and puddling, transplanting, and weeding (Bhandari et al., 2015; Dhital, 2017). Labor scarcity during crop establishment time prolongs the age of seedlings and transplantation of old seedlings affect rice productivity (Liu et al., 2017). Therefore, mechanization in rice production is crucial to reduce the cost of production and accomplish timely crop establishment and other inter-cultural operations. Rice contributes about 21% to the agriculture gross domestic product (AGDP). Per capita rice consumption in Nepal is around 134 kg (CBS, 2015).

Nepal currently imports cereal grains equivalent to US$386 million per year, and most of those imports are rice from neighboring India (GoN, 2017). Technological interventions that enhance productivity, and decrease the cost of production are likely to minimize trade deficits while improving food security. This paper addresses the emerging key policy questions by accompanying a comprehensive *ex-post* assessment of mini-tiller adoption on rice productivity at the household level in the hilly areas of Nepal. In particular, the following key policy questions are addressed: (1) what are the factors that govern the adoption of the mini-tiller in the mid-hills of Nepal?; (2) what is the productivity impact of mini-tiller adoption on rice cultivation?; and (3) does adoption of a mini-tiller improve the productivity of very small rice producing farms?

## 3 Materials and methods

### 3.1 Data

The current study is based on primary household survey data collected from the mid-hills of Nepal. Data were collected through a face-to-face interview with a structured questionnaire deployed on electronic software surveybe (http://surveybe.com, last assessed 9/10/2018) in order to minimize the data entry errors. Several skips and validation rules were applied in the software to minimize the time of the survey. The questionnaire elicited information on household socio-economic status, cropping systems, income sources, and inputs and outputs for rice production.

**Fig. 2:**
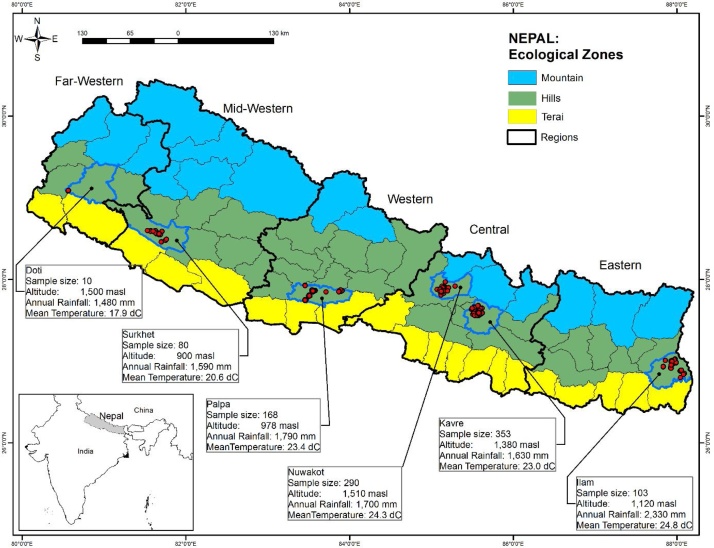
Map shows the sampled districts and samples (red dots) distribution in the mid-hills of Nepal.

The survey was conducted from October to November 2017, immediately following rice harvest season. The sampling frame encompassed six districts across the mid-hills of Nepal.^2^ These districts were selected based on the highest number of mini-tillers sold by private sector machinery traders from data provided by the Nepal Agricultural Machinery Entrepreneurs Association (NAMEA). After consultation with the District Agriculture Development Officers and key informants in each district, a total of 39 sub-districts (Village Development Committees: VDCs) from the six districts were selected purposively based on the higher density of mini-tillers. Finally, a pool of 1004 households were selected randomly from the sampled VDCs that consisted of 376 mini-tiller adopters and 628 non-adopters. However, only 624 households (62.15%) grew rice during the survey year, and these rice-growing households' data are used for this study. In this study, mini-tiller adopters are defined as the farmers who owned mini-tillers and used for rice cultivation and/or farmers who rented mini-tillers for rice cultivation. Out of 624 rice cultivating farms, 210 farms (33.65%) were mini-tiller owner adopters, and 92 farms (14.74%) were the adopter renters (who rented in the mini-tiller services). When we tested the difference in rice productivity between mini-tiller owner adopters and mini-tiller renters, no significant difference in rice productivity was detected, and hence we aggregated both groups of adopters (i.e., owner adopters and renter adopters) and defined them as mini-tiller adopters in this study. The non-adopters are the farm household who did not use the mini-tiller for rice cultivation instead they depend on traditional bullocks, laborers, and animal tractions for their rice land preparation. The distribution of selected districts and samples are presented in Fig. 2.

### 3.2 Empirical framework

We assumed that profit-maximizing and economically-rational farmers adopt a technology if the expected net benefit from its adoption is greater than the benefit without adoption (Mishra et al., 2018a, 2018b, 2017a, 2017b; Min et al., 2017; Teklewold and Mekonnen, 2017). Let us assume that $\hat{Y}$ is the difference in net gain in rice productivity between mini-tiller adopters and non-adopters. Then, if $\;\hat{\;Y}>0$, it implies that the adoption of a mini-tiller is more beneficial to the farmer than non-adoption. However, $\hat{Y}$ is unobservable but can be expressed as the function of observed farm-level socio-economic attributes in a latent model and can be presented as:

(1) Yˆ=βXi+εi, τ=1, if Yˆ>00, otherwise

Where, $\tau_{i}$ is a binary indicator variable that takes a value of 1 if household i adopts a mini-tiller and 0 otherwise. β is the vector of parameters to be estimated, $X_{i\;}$ is a vector of farm-level socio-economic attributes that determine mini-tiller adoption, and $\varepsilon_{i}$ is the error term which is assumed to be normally distributed. In the above described framework of Eq. (1), estimating the causal effect of technology adoption on outcome indicators is difficult due to the likelihood of an endogeneity problem. Estimating the true causal effect of technology adoption requires controlling observed and unobserved heterogeneity between technology adopters and non-adopters (Wooldridge, 2010).

Technology adopters and non-adopters may differ in their inherent farm abilities, managerial skills, individual working efficiencies, and their perceptions. Failure to account for such unobserved and observed heterogeneity may confound causal analyses and methods are required that account for group heterogeneity. In this study, we used an endogenous switching regression (ESR) approach for this purpose, following the literature (Mishra et al., 2018a, 2018b, 2017a, 2017b). To apply ESR, an instrument is required that affects rice productivity through mini-tiller adoption. In some of the adoption and impact literature, household caste has been used as an instrument (Kumar et al., 2018). Following Kumar et al. (2018), we also used general caste as an instrument, and it is plausible to assume that households being affiliated with a particular caste cannot affect rice productivity without the adoption of technology.^3^

We established the admissibility of the instrument by performing a simple falsification test following Di Falco et al. (2011). If an instrument is valid, this affects the adoption of mini-tillers; however, it will not influence the outcome variable (i.e., rice productivity) for the mini-tiller non-adopters (Table 1). After a suitable instrument is identified, the ESR addresses the problem of endogeneity by estimating the selection equation (first stage) and the outcome equation (second stage) simultaneously, using the full information for maximum likelihood calculations (Lokshin and Sajaia, 2004).

**Table 1:** Parameter estimates – validity test for selection instrument.

Parameter estimates	Dependent variable (1=Mini-tiller adoption)		Log of rice yield (kg/ha) among non-adopters	
	Coefficient	Std. error	Coefficient	Std. error
Constant	−0.512***	0.079	8.450***	0.049
General caste	0.828***	0.104	−0.032	0.031
Wald test on instrumental variable	$\chi^{2}$= 64.23		F-stat = 0.44	
No of observations	624		322	

*** significant at 1% level.

Given the conceptual framework as described above, the outcome function, conditional on adoption, can be specified as an ESR model in the following ways:

(2) $$Regime1\;:\;Y_{1i}=f\left(MT,\;X,\;\beta_{1}\right)+\varepsilon_{1i},if\;\tau_{i}=1$$

(3) $$Regime2\;:\;Y_{2i}=f\left(X,\;\beta_{2}\right)+\varepsilon_{2i},\;if\;\tau_{i}=0$$

Where $Y_{1i}$ represents outcome indicator (rice productivity) for mini-tiller adopters and $Y_{2i}$ for non-adopters; $\varepsilon_{i}$ is the error term of the outcome variables. The variable MT represents the adoption of mini-tillers, while X represents a household's socio-economic and farm-level attributes. $\beta_{1}$ and $\beta_{2}$ are the vector of parameters to be estimated that determines the outcome indicators for mini-tiller adopters and non-adopters, respectively. Finally, the variable $\tau_{i}$ measures adoption status ($\tau_{i}=1$, implies that the farmer is an adopter, 0= otherwise). The error term in the selection Eq. (1) and in outcome Eqs (2) and (3) are assumed to have a tri-variate normal distribution with mean zero and covariance matrix $\left(\Omega \right)$ in the following way:

$$\Omega =\left(\begin{array}{ccc} \sigma_{\mu }^{2} & \sigma_{1\mu } & \sigma_{2\mu }\\ \sigma_{\mu 1} & \sigma_{1}^{2} & .\\ \sigma_{\mu 2} & . & \sigma_{2}^{2} \end{array}\right)$$

where, ${\;\sigma }_{\mu }^{2}=var(\mu_{i})$, $\;\sigma_{1}^{2}=var(\varepsilon_{1})$, ${\;\sigma }_{2}^{2}=var(\varepsilon_{2})$, $\;\sigma_{1\mu }=cov(\mu_{i},\;\varepsilon_{1})$, ${\;\sigma }_{2\mu }=cov\left(\mu_{i},\;\varepsilon_{2}\right).$ Further, $\sigma_{\mu }^{2}$ is estimable up to a scale factor and can be assumed to have a value of 1 (Maddala, 1983). Additionally, if the correlation between the error term in the selection equation and the outcome equation is not equal to zero ($corr\left(\mu_{i},\;\varepsilon_{1}\right)\neq 0\;and\;corr(\mu_{i},\;\varepsilon_{2})\neq 0$), then it indicates the existence of selection bias (Lokshin and Sajaia, 2004). ESR addresses the selection bias by estimating the inverse Mills ratios $\;(\lambda_{1i}\;and$ $\lambda_{2i})$ and the covariance terms ($\sigma_{1\mu }\;and\;\sigma_{2\mu })$ and by including them in an auxiliary regression in Eqs. (2) and (3). If $\sigma_{1\mu }\;and\;\sigma_{2\mu }$ are significantly different from zero, then the absence of selection bias is rejected (Lokshin and Sajaia, 2004). The ESR model estimates can then be used to estimate ATT (average treatment effect on the treated) households as:

(4) $$E(Y_{1i}|\tau_{i}=1)=f\left(MT,\;X,\;\beta_{1}\right)+\lambda_{1i}\sigma_{1\mu }$$

(5) $$E(Y_{2i}|\tau_{i}=0)=f\left(MT,\;X,\;\beta_{2}\right)+\lambda_{2i}\sigma_{2\mu }$$

(6) $$E(Y_{2i}|\tau_{i}=1)=f\left(MT,\;X,\;\beta_{2}\right)+\lambda_{1i}\sigma_{2\mu }$$

(7) $$E(Y_{1i}|\tau_{i}=0)=f\left(MT,\;X,\;\beta_{1}\right)+\lambda_{2i}\sigma_{1\mu }$$

The average treatment effect on the treated (ATT) is then defined as the difference between Eq. (4) and Eq. (6) and can be written as:

(8) $$ATT=E(Y_{1i}|\tau_{i}=1)-E(Y_{2i}|\tau_{i}=1)$$

Similarly, the average treatment effect on the untreated (ATU), for the households that did not adopt mini-tillers, is the difference between Eqs (7) and (5). This captures the difference between what non-adopters would have benefited from had they adopted mini-tillers, and the observed rice productivity they obtained without adoption. The ATU can then be expressed as:

(9) $$ATU=E(Y_{1i}|\tau_{i}=0)-E(Y_{2i}|\tau_{i}=0)$$

## 4 Results and discussion

### 4.1 Descriptive analysis

**Fig. A1:**
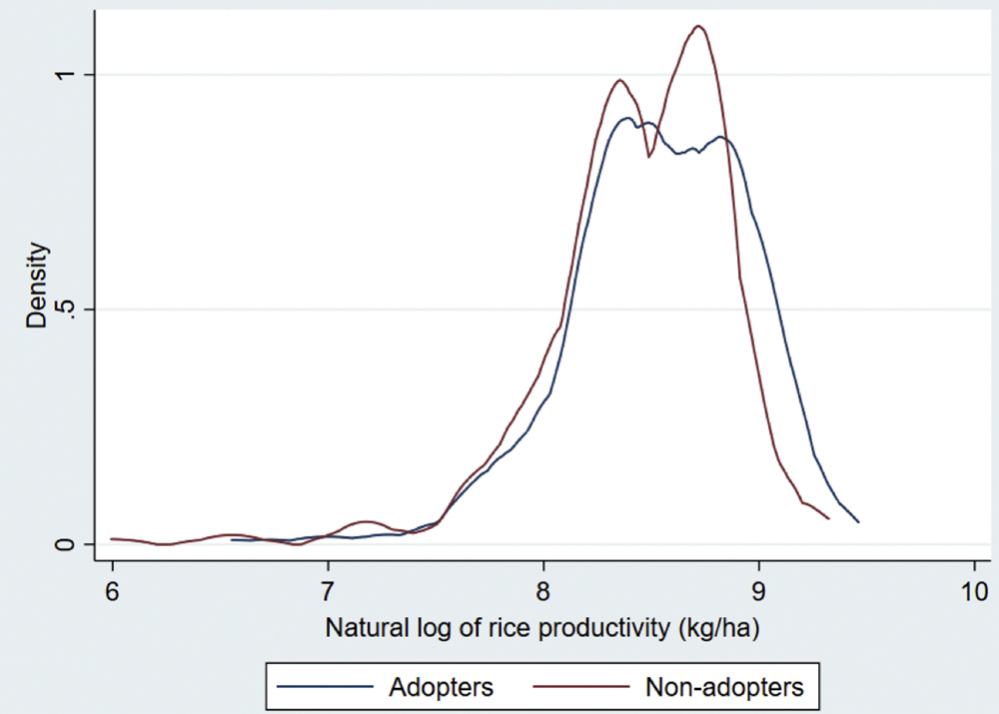
Distribution of rice productivity for mini-tiller adopters and non-adopters in the mid-hills of Nepal.

The descriptive statistics of the variables for the overall farms, mini-tiller adopters, and non-adopters are presented in Table 2. Almost half (302 households; 48%) of the rice-growing farmers interviewed were mini-tiller adopters.^4^ It was observed that sample households cultivate rice in an area of 0.38 ha. At 0.45 ha, mini-tiller non-adopters have a 30% smaller rice area than the adopters. In addition, the seed and fertilizer costs per unit area for mini-tiller non-adopters are significantly lower than adopters. However, the investment in capital and labor cost is significantly higher for the non-adopters. Rice productivity for all farms in the sampled districts is about 5245 kg per hectare, and the mini-tiller non-adopters have almost a 10% lower rice productivity than adopters. The distribution of the rice productivity for mini-tiller adopters and non-adopters is presented in the Appendix (Fig. A1).

**Table 2:** Inputs and outputs relationship for rice production with and without mini-tiller adoption.

Variables	Full sample (N = 624)		Adopters (N = 302)		Non-adopters (N = 322)		Difference (%)
	Mean	Std. error	Mean	Std. error	Mean	Std. error	
Rice productivity (kg/ha)	5245.17	79.25	5517.81	121.76	4989.46	100.81	−9.58***
Rice area (ha)	0.38	0.01	0.45	0.02	0.31	0.01	−30.41***
Seed cost (NPR/ha)	5186.20	157.48	5540.51	233.56	4853.89	211.16	−12.39**
Fertilizer cost (NPR/ha)	5772.84	243.98	7261.11	407.31	4377.01	255.81	−39.72***
Labor cost (NPR/ha)	53,032.16	1582.82	47,899.28	2027.95	57,846.23	2378.39	20.77***
Capital (NPR/ha)	34,363.58	829.32	26,972.46	1011.40	41,295.62	1173.80	53.10***
Total variable cost (NRP/ha)	98,354.77	2,089.97	87,673.36	2,587.03	108,372.70	3,145.85	23.61***
Gross revenue (NPR/ha)	159,660.40	2,996.83	166,572.70	4,636.25	153,177.40	3,821.52	−8.04**
Gross profit (NPR/ha)	61,305.61	3,598.31	78,899.36	4,947.86	44,804.64	5,041.31	−43.21***

*** significant at 1% level.

** significant at 5% level. Exchange rate: 1 US $ = NPR 104, during the survey year 2017 (NRB, 2018).

Mini-tiller adopters and non-adopters differ in many aspects of household socio-economic and farm-level attributes (See Table 3). The average farm size for all farm households is 0.51 ha; however, the non-adopters owned 26% smaller farms than adopters.^5^ Households with more family members, with a male as the main decision maker, who are younger in age, and with a higher level of education form the majority of the adopters.

**Table 3:** Attributes of mini-tiller adopters and non-adopters for rice production in the mid-hills of Nepal.

Variables	Full sample (N = 624)		Adopters (N = 302)		Non-adopters (N = 322)		Difference (%)
	Mean	Std. error	Mean	Std. error	Mean	Std. error	
*Demographic*							
Age of household head (years)	48.23	0.44	49.34	0.61	47.19	0.63	−4.36***
Household size (no)	5.91	0.09	6.10	0.13	5.73	0.11	−6.11**
Gender of household head (1=male, 0=female)	0.85		0.92		0.78		−14.68***
Caste of household (1=general caste, 0=others)	0.56		0.73		0.41		−43.47***
*Human capital*							
Education of household head (years)	5.83	0.18	6.87	0.26	4.85	0.24	−29.35***
Farming experience (years)	25.34	0.47	25.95	0.68	24.77	0.66	−4.54
Occupation of household head (1=farming, 0=others)	0.64		0.65		0.62		−3.82
*Land and livestock assets*							
Farm size (ha)	0.51	0.02	0.59	0.03	0.44	0.02	−25.57***
No of livestock holding (TLU)^#^	2.20	0.05	2.27	0.08	2.14	0.07	−5.74
Bullock availability (1=difficult, 0=easy)	0.28		0.44		0.13		−71.09***
*Household assets*							
Own mobile phone (1=yes, 0=no)	0.96		0.98		0.93		−4.63***
Own television (1=yes, 0=no)	0.94		0.98		0.89		−9.05***
Own engines such as pumps (1=yes, 0=no)	0.36		0.49		0.23		−53.42***
Off-farm income (‘000 NPR)	292.55	10.66	306.71	18.13	279.28	117.10	−8.94
*Access to facilities*							
Nearest input market distance (km)	8.43	0.36	3.56	0.22	13.00	0.55	264.77***
No of household members migrated (no)	0.33	0.02	0.26	0.03	0.38	0.03	44.22***
Credit access (1=yes, 0=no)	0.96		0.99		0.94		−4.32***
Group/cooperative members (1=yes, 0=no)	0.75		0.84		0.66		−21.41***
*Farm Inputs*							
On-farm labor wage rate (NPR/day)	692.52	8.29	695.03	12.38	690.16	11.12	−0.70
NPK fertilizer applied (kg/ha)^##^	85.82	3.54	109.28	6.00	63.82	3.51	−41.60***
Farmyard manure applied (1=yes, 0=no)	0.61		0.50		0.71		42.24***
Used improved rice variety (1=yes, 0=no)	0.13		0.13		0.12		−3.81
Used hybrid rice variety (1=yes, 0=no)	0.61		0.67		0.56		−15.96***
Grow spring rice (1=yes, 0=no)	0.08		0.12		0.03		−72.12***
Irrigation status (1=irrigated, 0=not irrigated)	0.94		0.96		0.92		−4.26**

*** significant at 1% level.

** significant at 5% level. Exchange rate: 1 US $ = NPR 104 during the survey year 2017 (NRB, 2018). ^#^TLU stands for tropical livestock unit (Pica-ciamarra et al., 2007). ^##^NPK indicates the amount of nitrogen, phosphorus, and potash applied through different forms of fertilizers (e.g., urea, DAP, and MoP).

The difference in mini-tiller adoption is reflected in terms of household assets as well as social and financial capital. Adopters have more land and livestock, and a higher percentage own a television, mobile phones, and irrigation water pumps. They also have a higher level of access to formal credit services and are more involved in groups and cooperatives. On the other hand, input market distance and the number of household members who had migrated were significantly higher for the farms in the non-adoption category.

The differences in farm-level attributes for adopters are also reflected in the higher rates of mineral fertilizer application and less farmyard manure applied to rice. Mini-tiller use rates were significantly higher in areas where farmers used hybrid rice varieties, and rice fields are irrigated. Although no significant difference in on-farm labor wage rates was detected across adoption categories, a higher portion of adopter farmers reported difficulty in accessing draft animals (bullocks) for field preparation. These differences between mini-tiller adopters and non-adopters necessitate the use of ESR that accounts for observed and unobserved heterogeneity.

### 4.2 Determinants of mini-tiller adoption

The ESR approach employed jointly estimate the selection equation in the first stage and the outcome equation in the second stage. The empirical results on the selection equation can be interpreted as normal probit coefficients. The coefficient value of $\rho_{1\mu }$ and $\rho_{0\mu }$ as shown in the selection equation of Table 4 represents the correlation of error terms in the selection equation and the outcome equation. The coefficient of $\rho_{1\mu }$ is positive and statistically significant, while the coefficient of $\rho_{0\mu }$ is statistically non-significant. The significant coefficient value of $\rho_{1\mu }$ in the selection equation indicates the presence of selection bias and, hence, it was appropriate to use an endogenous switching regression rather than ordinary least squares regressions. The ESR results for rice productivity are presented in Table 4.

**Table 4:** Endogenous switching regression estimates for rice productivity in the mid-hills of Nepal.

Variables	Selection equation		Log of rice yield (kg/ha)			
			Adopters		Non-adopters	
	Coefficient	Std. error	Coefficient	Std. error	Coefficient	Std. error
Constant	−5.492***	0.984	8.402***	0.403	7.279***	0.207
*Demographic*						
Age of household head (years)	0.006	0.013	−0.004	0.004	0.008**	0.004
Household size (no)	0.102**	0.045	−0.023**	0.011	−0.013	0.013
Gender of household head (1=male)	0.185	0.245	−0.042	0.085	0.019	0.056
Caste of household (1=general caste)	0.342**	0.154				
*Human capital*						
Education of household head (years)	0.082***	0.022	0.001	0.007	0.028***	0.007
Years of farming (years)	−0.001	0.011	0.010***	0.003	0.002	0.004
Occupation of household head (1=farming)	0.091	0.170	−0.041	0.052	−0.008	0.047
*Land & livestock assets*						
Farm size (ha)	0.026	0.164	−0.173***	0.050	0.013	0.050
No of livestock holdings (TLU)	−0.021	0.061	0.022	0.016	−0.026	0.018
*Household assets*						
Mobile phone holdings (1=yes)	−0.202	0.429	−0.326***	0.158	0.032	0.083
Television owning (1=yes)	0.737**	0.383	−0.024	0.177	−0.051	0.073
Household owning engines (1=yes)	0.375**	0.158	0.057	0.048	−0.124**	0.052
Off-farm income (NPR)	−4E-07	3E-07	−1E-09	9E-08	−9E-09	1E-07
*Access to facilities*						
Nearest market distant (km)	−0.239***	0.024	−0.008	0.009	0.005	0.005
Household members migrated (no)	−0.057	0.158	0.015	0.052	−0.048	0.040
Credit access (1=yes)	0.598	0.576	−0.010	0.200	−0.098	0.097
Group/cooperative members (1=yes)	0.110	0.200	−0.155**	0.065	−0.023	0.048
*Farm inputs*						
Log of NPK fertilizer applied (kg/ha)	−0.015	0.020	0.001	0.006	−0.006	0.005
Farm yard manure applied (1=yes)	0.109	0.162	−0.074	0.048	−0.006	0.046
Bullock availability (1=difficult)	0.742***	0.170	0.012	0.051	0.166***	0.065
On-farm labor wage rate (NPR/day)	0.004***	0.001	3E-04*	2E-04	3E-04*	2E-04
Use improved rice variety (1=yes)	−0.172	0.251	0.170**	0.077	−0.003	0.069
Use hybrid rice variety (1=yes)	−0.011	0.183	0.214***	0.058	0.212***	0.047
Grow spring rice (1=yes)	2.114***	0.483	−0.057	0.084	−0.060	0.113
Irrigation status (1=irrigated)	0.797***	0.281	0.458***	0.113	0.534***	0.082
${ln\sigma }_{1}$			−0.989***	0.048		
$\rho_{1\mu }$			0.392*	0.219		
${ln\sigma }_{0}$					−1.073***	0.043
$\rho_{0\mu }$					0.287	0.232
No of observations	624		302		322	
Wald $\chi^{2}$	105.59					
Log-likelihood	−428.776					

^***^significant at 1% level; **significant at 5% level; *significant at 10% level. Exchange rate: 1 US $ = NPR 104 during the survey year 2017 (NRB, 2018).

Farm households with larger families, more years of formal schooling, owning televisions and pumps, and located closer to the input markets tend to adopt the mini-tillers. Also, households from the general caste were likely to adopt mini-tillers compared to other castes such as the *dalit*, *janjati*, etc. Furthermore, farm households who face difficulties in finding bullocks for agricultural purposes and had to pay higher on-farm labor wages due to labor shortages have a higher probability of adopting mini-tillers. Finally, farms having more irrigated rice land and growing spring rice tend to adopt mini-tillers.

The issue of high labor prices and bullock scarcity are intertwined due to the higher cost of keeping livestock (FAO, 2010; Kumar et al., 2017; Maharjan et al., 2013a; Pradhanang et al., 2015).^6^ It should be noted that livestock productivity in Nepal is the lowest among South Asian countries due to insufficient fodder, grazing lands, and feed (Rao and Birthal, 2008). The percentage of draft animals in the herd is also decreasing due to emerging market-oriented dairy opportunities that provide a more remunerative allocation strategy for feeding resources (Upadhyay et al., 2017).

The estimates in the outcome equations in the columns for adopters and non-adopters of Table 4 represent the determinants of rice productivity for the mini-tiller adopters and non-adopters. Farm size is negatively associated with rice productivity, indicating a higher productivity for small farms than for large farms. However, coefficients for improved varieties, hybrid varieties, and irrigation are statistically significant and positively associated with the rice productivity of mini-tiller adopters. Therefore, the adoption of improved and hybrid rice varieties increased rice productivity by 0.170% and 0.214%, respectively for the mini-tiller adopters. Similarly, the irrigation of rice increased rice productivity by 0.458%. Finally, although the signs of the coefficient of fertilizers was positive for adopters and negative for non-adopters, no significant effect on rice productivity was detected.

### 4.3 Does mini-tiller adoption improve rice productivity?

The results from the mini-tiller adoption impact assessment are presented in Table 5. The ATT (average treatment effect on the treated) in Table 5 shows the difference between what the rice productivity adopters have gained after mini-tiller adoption and without mini-tillers adoption. ATU (average treatment effect on the untreated) is the difference between non-adopters observed productivity and if non-adopters had adopted mini-tillers. The results show that the adoption of the mini-tiller has a significant and positive impact on rice productivity (ATT = 1110 kg/ha).^7^ Furthermore, if the mini-tiller adopters had not adopted mini-tillers, their rice productivity would have decreased by 27%, and if the non-adopters had adopted the mini-tillers, their rice productivity would have increased by 1250 kg/ha (26%).

**Table 5:** Impacts of mini-tiller adoption on rice productivity in the mid-hills of Nepal.

Outcome indicator	Farm household types	Decision to		Treatment effect	Change
		Adopt	Not to adopt		
Rice productivity (kg/ha)	Adopters (ATT)	5204 (61)	4094 (53)	1110*** (62)	27.11%
	Non-adopters (ATU)	6021 (104)	4771 (67)	1250*** (87)	26.20%

*** significant at 1% level. ATT: Average treatment effect on the treated. ATU: Average treatment effect on the untreated. Numbers in parenthesis indicate standard errors.

Increasing labor wages due to labor shortages are the consequences of labor out-migration in Nepal (Tuladhar et al., 2014). Due to the labor shortage and rising wages, farmers are unable to manage the crop cultivation practices in time (Maharjan et al., 2013a, 2013b) and the effects are more serious for labor-intensive crops such as rice. Farmers in Nepal have started to leave the cultivated land as fallow due to the high cost of production associated with farming (Khanal, 2018). Since wage rates are considered as a proxy indicator for the labor shortage (Zhang et al., 2014, 2011) and our study has demonstrated a positive association between mini-tiller adoption and labor wages, the adoption of mini-tillers has attenuated labor shortages for crop establishment. Hence, the adoption of mini-tillers has increased rice productivity significantly in the mid-hills of Nepal, which contributes to household food security in the area.^8^

In order to understand which types of farms have the highest probability of a positive impact of mini-tiller adoption on outcome indicator, the sample data were stratified into four quartiles of farm size. The rice productivity effects of mini-tiller adoption across different farm size quartiles are shown in Table 6. The results show that the adoption of mini-tillers has a significant positive impact on rice productivity for all farms in the four quartiles. However, despite having qualitatively similar impacts, the magnitude of the impact of mini-tiller adoption across farm-size quartiles differed. The smallest quartile farms of ≤0.25 ha have the highest gain in rice productivity (1535 kg/ha; 36%) with mini-tiller use.^9^ Furthermore, the non-adopters in the smallest quartile farms (≤2.05 ha) could have gained 34% more rice productivity had they adopted mini-tillers. These results indicate that the smallest and most marginal farmers were most benefited from mini-tiller adoption.

**Table 6:** Impacts of mini-tiller adoption on rice productivity across farm quartiles in the mid-hills of Nepal.

Farm size quartiles^#^	Farm household sub-samples	Rice productivity (kg/ha)			
		To-adopt	Not to adopt	Treatment effect	Change
First quartile (≤0.25 ha)	Adopters (ATT)	5746 (195)	4210 (149)	1535*** (194)	36.46%
	Non-adopters (ATU)	6764 (171)	5055 (114)	1709*** (145)	33.81%
Second quartile (0.25 – 0.40 ha)	Adopters (ATT)	5586 (128)	4396 (122)	1191*** (133)	27.09%
	Non-adopters (ATU)	5920 (191)	4868 (137)	1052*** (165)	21.61%
Third quartile (0.40 – 0.60 ha)	Adopters (ATT)	5272 (86)	4132 (94)	1140*** (90)	27.59%
	Non-adopters (ATU)	5712 (195)	4483 (131)	1229*** (180)	27.41%
Fourth quartile (≥0.60 ha)	Adopters (ATT)	4713 (100)	3831 (82)	882*** (114)	23.02%
	Non-adopters (ATU)	4733 (181)	4311 (132)	422** (156)	9.79%

^#^Note: The quartiles are based on farm size. ***significant at 1% level; **significant at 5% level. ATT: Average treatment effect on the treated. ATU: Average treatment effect on the untreated. Number in parenthesis indicate standard errors.

### 4.4 Robustness check using matching methods

We checked the robustness of our findings by comparing the results from two alternate matching methods, namely propensity score matching (PSM), and inverse probability weighted regression adjustment (IPWRA). We followed Caliendo and Kopeinig (2005) to derive the PSM estimates, and we used STATA 15 (www.stata.com) inbuilt command to derive the IPWRA estimates and the results are presented in Table 7.^10^ Here, we were interested to know what could have been the rice productivity effects of mini-tiller adoption while considering the observed heterogeneity among adopters and non-adopters. The average treatment effect results from PSM and IPWRA are statistically significant, suggesting the positive impacts of mini-tiller adoption on rice productivity. Nevertheless, the impact magnitude of outcome indicators i.e., rice productivity is considerably lower than the result from ESR (PSM ATT = 688 kg/ha and IPWRA ATT = 899 kg/ha). It should be noted that these alternative matching methods used for robustness checks accounts for only the observed heterogeneity and do not account for unobserved heterogeneity. The underestimated results from PSM and IPWRA could be due to the effects of unobserved heterogeneity in the model and requirement of the large samples with substantial overlap between treatment and control groups; hence, the use of ESR was more appropriate. Similar results were reported in earlier studies indicating the superior performance of ESR over PSM (Abdoulaye et al., 2018; Tucker, 2010; Wossen et al., 2017).

**Table 7:** Robustness check using matching methods.

Outcome indicators	Propensity score matching – NNM^#^			IPWRA^##^		
	ATT	Std. error	Change over non-adoption	ATT	Std. error	Change over non-adoption
Rice productivity (kg/ha)	688.53**	345.08	9.74%	899.10***	194.91	17.56%
Other controls	Yes			Yes		
No of observations	624			624		

***significant at 1% level; **significant at 5% level. ATT: Average treatment effect on the treated. ^#^NNM: Nearest neighbor matching in which three nearest neighbors were matched with replacement and common support. ^##^IPWRA: Inverse-probability-weighted regression adjustment.

## 5 Conclusion and policy implications

The present study assessed the impacts of scale-appropriate mechanization in the form of the mini-tiller on rice productivity in the mid-hills of Nepal. We used the endogenous switching regression method to account for observed and unobserved sources of heterogeneity between technology adopters and non-adopters.

Our findings suggest that adoption of mini-tillers is driven by the rising labor-wage rates and an associated decline in draft animal availability. Wealthier households with a higher level of education that are located closer to the input markets and have access to irrigation have a higher probability of adopting mini-tillers. To foster more inclusive access to the technology, results highlight the importance of access to service provision in order for households with poorer social and economic capital in the mid-hills of Nepal to utilize the technology. Targeting more educated farmers and would-be service providers through social marketing may also increase the number of mini-tillers in the mid-hills of Nepal.

Our results confirm substantial productivity benefits associated with mini-tiller use. Adoption increased rice productivity by 1110 kg/ha. The analysis suggests that non-adopters would obtain similar gains with estimated rice productivity gain of 1250 kg/ha had they adopted mini-tillers. Additionally, very small farms (≤0.25 ha) tend to benefit the most from mini-tiller adoption in terms of rice productivity. The results reported here in this study confirmed the significant positive impacts of scale-appropriate mechanization on rice productivity in the smallholder farming systems in the hilly areas of Nepal. Government policies that stimulate investments accordingly are likely to have highly positive impacts on smallholder rice productivity and livelihoods outcomes within Nepal and in adjacent production ecologies in South Asia where labor and draft animal scarcity is undermining timely field operations.

## Conflict of interest

We declare to have no conflict of interest.

## Footnotes

1 In this study we have taken mini-tillers as a case of scale-appropriate mechanization. A mini-tiller is a 5-9 horsepower small tractor with rotating blades, basically used for land preparation (soil tillage, puddling, and line sowing when attached with one- or two-line seed drills). Mini-tillers in some areas are also used for lifting water from canals and underground sources to irrigate the plots when their engines are used as power sources. The details of mini-tiller technology can be found in Acharya et al. (2017).

2 The districts were selected purposively such that at least one district is captured from each developmental region on an east-to-west gradient. Nepal has five developmental regions and a total of 39 districts are located in the mid-hills. However, we selected two districts in the central developmental region due to the higher concentration of mini-tillers. The selected districts were Doti (far-west region), Surkhet (mid-west region), Palpa (western region), Kavre and Nuwakot (central region), and Illam (eastern region).

3 In the recent years, the caste based discriminations has weakened due to government priorities (Mosse, 2018), particularly through increased in development expenditures (e.g., equitable market access, extension services, and inputs) and it’s therefore reasonable to assume that farm households with particular caste identify are less likely to influence directly with crop productivity.

4 Since we have purposively selected the districts and sub-districts (VDCs) based on the higher concentration of mini-tillers, this mini-tiller adoption rate for rice farming may not be representative for the entire mid-hills of Nepal. Moreover, almost one fourth of the mini-tillers owner adopter purchased mini-tillers without any subsidy, while rest (almost three fourth) of the farmers obtained subsidy on mini-tillers that ranges in the range of 25% to 100%.

5 Farm size also includes area under other crops (e.g. maize, millet, vegetables, wheat, pulses, and legumes) including rice.

6 While the average cost of a pair of bullocks (draft animals) in the study area was around *NPR 35,000*, the cost of mini-tiller without subsidy ranges from *NPR 35,000* to *NPR 130,000* with a mean cost of *NPR 69,652* (depending on capacity of mini-tillers).

7 The difference between the gains in rice productivity among subsidized and non- subsidized mini-tiller adopter farmers was not statistically significant.

8 It should be noted that the average treatment effect for the treated (ATT) 1,110 kg/ha is the productivity effect on a per hectare basis. However, the net impact at the household level for rice-producing mini-tiller adopters is almost 497 kg (i.e., ATT* adopters rice area = 1,110*0.448) on average.

9 The sample size diminishes on stratifying mini-tiller owner adopters by subsidized and non-subsidized mini-tiller owners across farm size quartiles hence testing the significant difference on rice productivity gain across these cohorts was difficult. The mean productivity gain was higher for the farms located in second and third quartiles for non-subsidized mini-tiller adopters, while the gain in rice productivity was higher for the farms placed in first and second quartiles of farm size under subsidized mini-tiller adopter category.

10 We used “psmatch2” and “teffects ipwra” commands of STATA to estimate the rice productivity effects of mini-tiller adoption by propensity score matching and inverse probability weighted regression adjustment, respectively.
